# Study on Phase I Metabolic Processes and Metabolite Biomarker Identification of Synthetic Cannabinoids 5F-ADB-PINACA and 5F-ADBICA in Human Liver Microsomes and Zebrafish Model

**DOI:** 10.3390/molecules31020250

**Published:** 2026-01-12

**Authors:** Huan Li, Hui Zheng, Shihao Zhong, Yanbiao Zhao, Jiaman Lin, Hongliang Su, Zhenhua Qian, Yuanfeng Wang

**Affiliations:** 1 Institute of Evidence Law and Forensic Science, China University of Political Science and Law, Beijing 100088, China; 2 Drug Intelligence and Forensic Center, Ministry of Public Security, Beijing 100193, China; 3 Food and Drug Anti-Doping Laboratory, China Anti-Doping Agency, 1st Anding Road, Chaoyang District, Beijing 100029, China; 4 School of Investigation, People’s Public Security University of China, Beijing 100038, China; 5 School of Forensic Medicine, Shanxi Medical University, Jinzhong 030600, China

**Keywords:** synthetic cannabinoids, human liver microsome, temporal metabolic profiling, correlation analysis, metabolite biomarker

## Abstract

Synthetic cannabinoids (SCs) are a rapidly developing kind of novel psychoactive substance, frequently associated with acute intoxication and public health concerns. This study aimed to elucidate and compare the phase I metabolic pathways of two structurally related SCs, 5F-ADB-PINACA and 5F-ADBICA, using in vitro and in vivo models. Temporal metabolic profiling was performed to identify potential signature metabolites. Temporal abundance patterns and correlation cluster analysis of metabolites were analyzed to determine metabolite biomarkers. The two SCs were incubated with pooled human liver microsomes for 24 h and were also evaluated in vivo in zebrafish. Metabolite profiles were characterized using UHPLC-QE Orbitrap-MS. HLM analysis identified 21 5F-ADB-PINACA metabolites and 28 5F-ADBICA metabolites. Metabolites of 5F-ADBICA were detected for the first time in vitro and in a zebrafish model. Zebrafish studies confirmed the presence of all key metabolites observed in HLM. Comparative analysis of their metabolic pathways revealed differences in metabolism driven by structural differences between the indazole and indole cores. This is the first time that correlation analysis has been used in the temporal metabolic profiling of SCs. This study comprehensively characterized the metabolism of 5F-ADB-PINACA and 5F-ADBICA, identifying M13 (hydrolytic defluorination) as a potential metabolite biomarker for 5F-ADB-PINACA and M19 (hydrolytic defluorination) as a potential metabolite biomarker for 5F-ADBICA. The metabolic reactions of the main metabolites of the two synthetic cannabinoids are consistent. However, their metabolic processes (i.e., the overall metabolic pathways and temporal progression of these reactions) are different, which illustrates the metabolic similarity of structurally similar synthetic cannabinoids and the impact of different structures on the metabolic processes.

## 1. Introduction

Synthetic cannabinoids (SCs), as an important subgroup of new psychoactive substances (NPS), have emerged frequently in illicit global markets since their emergence, with multiple novel structural analogs reported and added to early warning systems each year [1]. According to the latest European Drug Report, SCs represent the most prevalent group of new psychoactive substances (NPS) [2]. In Europe, annual monitoring reports indicated that SCs are commonly distributed in the form of herbal blends, e-liquids, and powders, and there are even reports of seized prison paper, demonstrating a high degree of market adaptability and posing persistent public health challenges for international drug control efforts [3,4,5]. These products are often deceptively marketed as “legal and safe”, yet they have been repeatedly associated with acute intoxication events. Furthermore, intensified regulatory restrictions have driven illegal laboratories to continuously modify the chemical structures of SCs, generating novel structures beyond existing regulatory control [6]. Pharmacological studies have demonstrated that many SCs exhibit higher efficacy and potency at CB_1_ receptors than Δ^9^-tetrahydrocannabinol (THC), leading to stronger and less predictable clinical manifestations [7].

Because SCs are rapidly metabolized in the human body, the concentration of their parent compounds in blood and urine may be below the detection limit. This limitation is not universal, as it depends on factors such as the sensitivity of the mass spectrometer, the volume of the tested sample, the time elapsed between the incident or intoxication and sample collection, as well as the stability of the compounds. Consequently, the identification of characteristic metabolites plays a pivotal role in confirming exposure to SCs [6]. Human liver microsomes (HLM), which are enriched in key human phase I metabolic enzymes, have been widely used to simulate the major metabolic pathways of SCs within a short time frame [8]. Recent studies utilizing HLM have successfully identified various metabolites of SCs, including oxidative defluorination, N-dealkylation, and hydroxylation products, and have proposed potential urinary metabolite biomarkers for forensic confirmation in authentic human samples [9,10,11,12]. However, HLM-based detection methods still have limitations. They cannot assess extrahepatic metabolic contributions or inter-individual differences in enzyme activity and, therefore, still differ from real human urine samples [13].

Zebrafish, as an ethically accessible and cost-effective vertebrate model, have gained increasing attention in the research on the metabolism of SCs due to their highly conserved phase I metabolic enzymes, which are predominantly mediated by cytochrome P450 isoforms [14]. This similarity enables zebrafish to serve as a reliable in vivo metabolic model [15,16]. Notably, zebrafish studies have demonstrated high concordance with human results, both in the number of phase I metabolites produced and in the types of metabolic transformations, including oxidation, hydroxylation, and N-dealkylation. These findings suggest that zebrafish can effectively complement HLM-based studies, providing cross-validation of key metabolites [14,15,16,17].

Previous studies on indole- and indazole-based SCs have revealed diverse metabolic pathways, including oxidative defluorination of fluorinated side chains, oxidation–reduction reactions, N-dealkylation, and hydroxylation occurring on both the core rings and the terminal side chains. In some cases, additional transformations, such as acetylation and defluorination to aldehyde, have also been reported [18,19,20,21,22]. High-resolution mass spectrometry has proven particularly critical for detecting trace-level diagnostic metabolites. For potent indazole SCs, such as 5F-MDMB-PICA and 4F-MDMB-BICA, hydroxylated metabolites have been identified as preferred urinary biomarkers [18]. However, comparative analyses of structurally similar compounds, such as 4F-MDMB-BICA and 4F-MDMB-BINACA, have demonstrated that even minor structural variations can shift the primary sites of metabolism, highlighting the limitations of analog-based predictions and underscoring the necessity of empirical metabolic studies for each emerging SC [19]. However, the study also shows that the “core structure” plays a crucial role in the metabolism of synthetic cannabinoids, and its key importance in determining their properties and other toxicological parameters can be identified. Ultra-high-performance liquid chromatography coupled with high-resolution mass spectrometry remains the gold standard for simultaneously capturing low- and high-abundance metabolites, thereby improving the reliability of biomarker identification.

Despite advances in metabolite characterization, significant gaps remain in our understanding of the temporal metabolic profiling of SCs. The majority of existing studies focus on short-term experiments aimed at identifying metabolite biomarkers but rarely investigate time-dependent variations in metabolite abundance. Comparative studies in mice have revealed compound-specific differences in pharmacodynamic duration among ADB-BICA, ADB-BINACA, ADB-4en-PINACA, and MDMB-4en-PINACA [23]. Furthermore, HLM-based investigations have demonstrated the feasibility of simulating time-resolved metabolic dynamics by tracking metabolite fluctuations within a 24 h incubation period, thereby facilitating the selection of stage-specific urinary markers [19,24]. However, comprehensive analyses of inter-metabolite relationships and temporal changes remain scarce and warrant further exploration.

This study focuses on two structurally related SCs, 5F-ADB-PINACA and 5F-ADBICA, both of which share an ADB-based tert-leucinamide scaffold and a 5-fluoropentyl side chain but differ in their indazole and indole cores, respectively. Although 5F-ADB-PINACA and 5F-ADBICA are not the latest synthetic cannabinoids, they and their structural analogues continue to appear in the global new psychoactive substances (NPS) market in recent years. Therefore, identifying their metabolite biomarkers remains important in analytical and forensic fields, particularly for toxicological screening, monitoring emerging analogues, and studying the metabolic behavior of structurally similar synthetic cannabinoids. While prior studies have investigated the metabolism of 5F-ADB-PINACA using human hepatocytes [25], no integrated analyses have combined both in vitro and in vivo models to comprehensively elucidate its metabolic profile. Moreover, to date, there have been no published reports on the metabolic fate of 5F-ADBICA. To address these gaps, we employed a combined approach integrating HLM-based in vitro assays, zebrafish-based in vivo modeling, and UHPLC-QE-Orbitrap-MS analysis to characterize the phase I metabolic pathways of these two compounds. Furthermore, we conducted time-resolved analyses of metabolite dynamics over 24 h and examined correlations among different metabolites to identify potential metabolite biomarkers. Through this comprehensive investigation, we aim to fill critical gaps in temporal metabolic profiling knowledge and provide a robust analytical framework for the forensic detection of 5F-ADB-PINACA and 5F-ADBICA. This study also provides a basis for future clinical studies on human intoxications.

## 2. Results and Discussion

### 2.1. Identification of 5F-ADB-PINACA Metabolites in HLM Incubation

**Figure 1:**
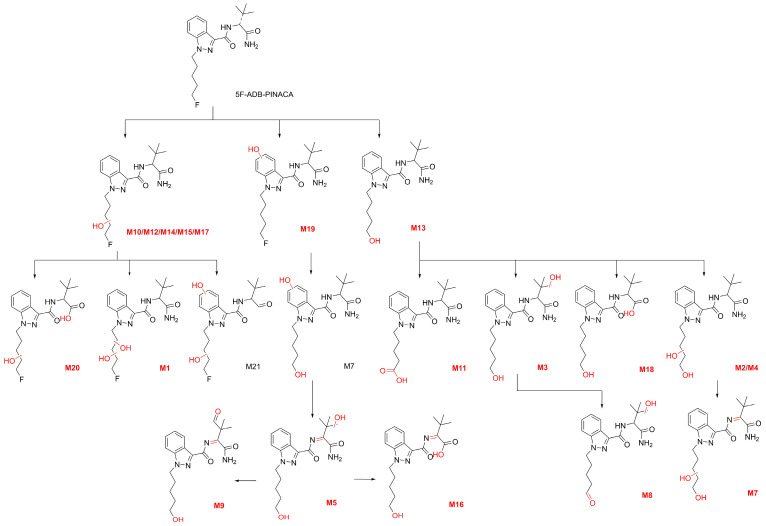
The 21 metabolites and predicted metabolic ways of 5F-ADB-PINACA. (The metabolites highlighted in red were detected in zebrafish).

A total of 21 metabolites of 5F-ADB-PINACA were detected in the HLM metabolic study. In this experiment, a total of eight metabolic reactions were discovered, including hydroxylation; dihydroxylation; dehydrogenation; amide hydrolysis; deamination; hydrolytic defluorination; oxidative defluorination to aldehyde; and defluorination to carboxylic acid. The metabolic reactions and corresponding reaction sites for each metabolite are detailed in Table 1. In addition, Table 1 provides the molecular formulas, retention times, exact [M+H]^+^ masses, and diagnostic product ions derived from secondary mass spectrometric analysis. All 21 structures of the metabolites and predicted metabolic processes are shown in Figure 1.

Compared to previous studies, this research significantly supplements the existing understanding of 5F-ADB-PINACA phase I metabolites. Previous studies identified relatively few phase I metabolites, involving only five metabolic reactions in total. This study comprehensively covers the 12 phase I metabolites discovered previously, identifying nine new phase I metabolites and entirely new metabolic reactions [25,26].

The following 21 metabolites are analyzed based on their different metabolic reactions. Due to the large number of metabolites, we group them according to all the metabolic reactions that occurred with each metabolite.

#### 2.1.1. Hydroxylation

During the metabolism of 5F-ADB-PINACA, a total of six hydroxylated metabolites were discovered, with their reaction sites located on the 5-fluoropentyl side chain and the indazole core. Five metabolites, M10, M12, M14, M15, and M17 ([M+H]^+^ *m*/*z* 379.2140), have their hydroxylation sites located on the 5-fluoropentyl side chain (Figures S1–S5). The product ion at *m*/*z* 145.0396 showed that no hydroxylation occurred on the core. So, the product ions *m*/*z* 231.0928, 249.1034, 267.1140, and 334.1925 indicated that these metabolites were hydroxylated on the 5-fluoropentyl side chain (the fragmentation pathway of M12, see Figure S6). Only M19 ([M+H]^+^ *m*/*z* 379.2140) exhibited hydroxylation on the indazole core, as indicated by the product ions at *m*/*z* 161.0346 and 179.0451 (Figure S7).

#### 2.1.2. Dihydroxylation

M1 ([M+H]^+^ *m*/*z* 395.2089) is the only dihydroxylated metabolite. Both hydroxylations occurred on the 5-fluoropentyl side chain. The product ions *m*/*z* 267.1140 and 145.0396 showed the dihydroxylation on the chain.

#### 2.1.3. Amide Hydrolysis + Hydroxylation

M20 ([M+H]^+^, *m*/*z* 380.1980) underwent amide hydrolysis on the ADB residue and concurrent hydroxylation on the 5-fluoropentyl side chain, as evidenced by the product ion at *m*/*z* 362.1874 and the diagnostic ions at *m*/*z* 251.1190 and 145.0396.

#### 2.1.4. Dihydroxylation + Deamination

M21 ([M+H]^+^ *m*/*z* 380.1980) was the metabolite with both dihydroxylation and deamination. Although this metabolite has the same chemical formula as M20, the product ion at *m*/*z* 267.1140 indicated that it was dihydroxylated on both the side chains and the indazole core. Furthermore, the product ion at *m*/*z* 161.0346 confirmed hydroxylation on the indazole core.

#### 2.1.5. Hydrolytic Defluorination

**Figure 2:**
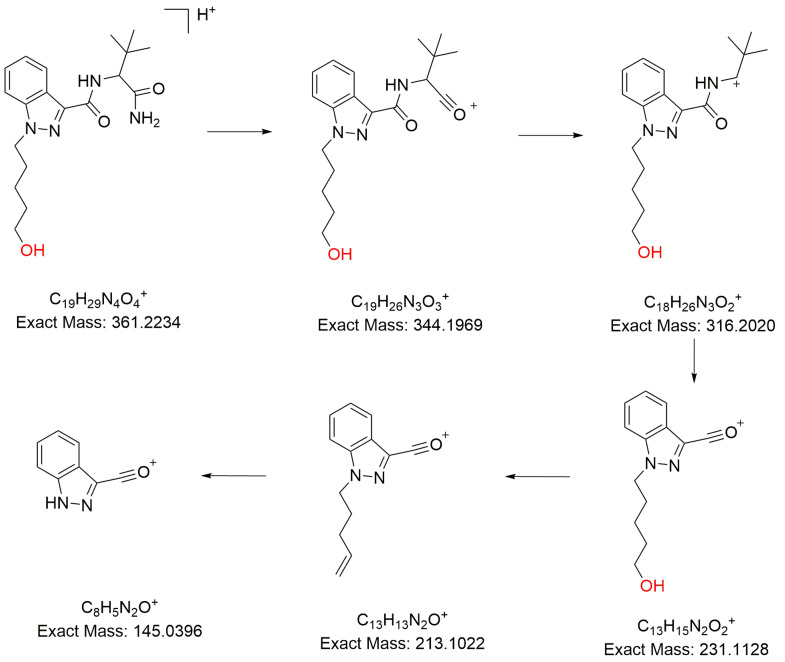
The fragment pathways of M13 of 5F-ADB-PINACA.

M13 ([M+H]^+^ *m*/*z* 361.2234) (Figure S8) was identified as the metabolite with hydrolytic defluorination. The chemical formula and the product ions at *m*/*z* 231.1128, 316.2020, and 344.1969 indicated that the fluorine on the 5-fluoropentyl side chain was replaced by a hydroxyl group (for the fragmentation pathway of M13, see Figure 2).

#### 2.1.6. Hydrolytic Defluorination + Hydroxylation

Four metabolites with hydrolytic defluorination and hydroxylation were identified. However, the hydroxylation reaction site of these four metabolites is different. For M2 and M4 ([M+H]^+^ *m*/*z* 377.2183), the product ions *m*/*z* 229.0972, 247.1077, and 332.1918 showed they were hydroxylated on the 5-fluoropentyl side chain (Figures S9 and S10). Conversely, the product ions *m*/*z* 213.1022 and 231.1128 showed M3 ([M+H]^+^ *m*/*z* 377.2183) was hydroxylated on the ADB residue (for the fragmentation pathway of M3, see Figures S11 and S12). But in M6 ([M+H]^+^ *m*/*z* 377.2183), the product ions *m*/*z* 161.0346 and 179.0451 indicated that the hydroxylation occurred on the indazole core (Figure S13).

#### 2.1.7. Hydrolytic Defluorination + Amide Hydrolysis

Amide hydrolysis occurred on the ADB residue of M18 ([M+H]^+^ *m*/*z* 362.2074), while hydrolytic defluorination also occurred on the 5-fluoropentyl side chain (Figure S14). The chemical formula and product ion *m*/*z* 362.2074 indicated the amide hydrolysis on the ADB residue. Furthermore, the product ions *m*/*z* 213.1022 and 231.1128 showed that the fluorine on the 5-fluorine 5-fluoropentyl side chain was hydrolyzed.

#### 2.1.8. Hydrolytic Defluorination + Dehydrogenation + Hydroxylation

Two metabolites with hydrolytic defluorination + dehydrogenation + hydroxylation were detected in this study. However, the reaction site of hydroxylation of two metabolites are different from each other. The product ions *m*/*z* 213.1022, 231.1128, and 357.1921 showed that the ADB residue of M5 ([M+H]^+^ *m*/*z* 375.2027) was hydrolyzed (Figure S15), and the product ions *m*/*z* 229.0972 and 245.0921 indicated the hydroxylation site of M7 was on the 5-fluoropentyl side chain.

#### 2.1.9. Oxidative Defluorination to Aldehyde + Hydroxylation

M8 ([M+H]^+^ *m*/*z* 375.2027) was identified as the metabolite with defluorination to aldehyde and hydroxylation. The product ion *m*/*z* 201.1022 showed the oxidative defluorination to aldehyde reaction on the 5-fluoropentyl side chain. In addition, the product ions *m*/*z* 145.0396, 330.1812, and 358.1761 showed the ADB residue was hydrolyzed.

#### 2.1.10. Hydrolytic Defluorination + Dehydrogenation + Oxidation to Aldehyde

M9 ([M+H]^+^ *m*/*z* 373.1870) was identified as a metabolite with hydrolytic defluorination, dehydrogenation, and oxidation to aldehyde. The product ions *m*/*z* 213.1022 and 231.1128 showed the fluorine on the 5-fluoropentyl side chain was hydrolyzed. In addition, comparison of the [M+H]^+^ ion of M9 with that of the parent compound suggested that the degree of unsaturation of the ADB residue increased by two. An aldehyde group was, therefore, inferred to be formed on the butyl moiety of the ADB residue, accompanied by dehydrogenation of the ADB group. This observation confirms previous studies showing that ketones can be formed during the metabolism of short alkyl chains [27].

#### 2.1.11. Hydrolytic Defluorination + Dehydrogenation + Amide Hydrolysis

M16 ([M+H]^+^ *m*/*z* 360.1918) was the metabolite with hydrolytic defluorination + dehydrogenation + amide hydrolysis. The product ions *m*/*z* 213.1022 and 231.1128 showed the fluorine was hydrolyzed. Based on the chemical formula and the [M+H]^+^ ion of M16, dehydrogenation and amide hydrolysis on the ADB residue were confirmed (Figure S16).

#### 2.1.12. Defluorination to Carboxylic Acid

M11 ([M+H]^+^ *m*/*z* 375.2027) stands as the sole metabolite undergoing carboxylation (the MS2 spectrum of M11 is shown in Figure S17). The product ions *m*/*z* 217.0972 and 245.0921 showed the fluorine was converted into a carboxylic acid (Figure S18).

### 2.2. Temporal Metabolic Profiling and Potential Metabolite Biomarker of 5F-ADB-PINACA

#### 2.2.1. Temporal Trends of 5F-ADB-PINACA Metabolites

**Figure 3:**
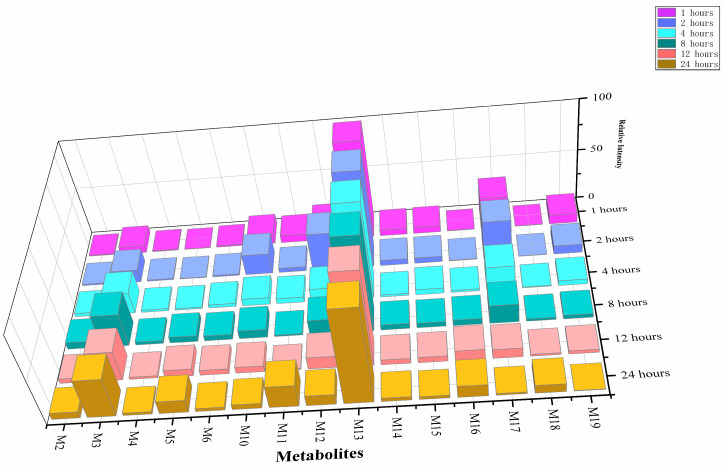
The relative intensity of the changes over time of 15 selected metabolites of 5F-ADB-PINACA.

After 24 h of incubation in HLM, 5F-ADB-PINACA produced 21 metabolites in vitro. To facilitate the study, we selected the 15 metabolites with the highest relative intensities. Figure 3 shows the relative intensities of these 15 metabolites, which changed significantly over time. M13 remained at the highest level throughout the incubation period and was the main metabolite. M3 continued to increase over time, becoming the second most abundant metabolite, second only to M13 at 24 h. M5, M16, and M18 also showed a slow increase. However, the increase was less pronounced than that of M13 and M3. In contrast, metabolites such as M10, M12, M6, and M4 reached their highest levels early in the experiment and then rapidly declined. M12 was the most representative metabolite, peaking at 2 h before experiencing a significant decline. Because the 24 h incubation likely exceeds physiological hepatic exposure, late-appearing metabolites should be interpreted cautiously, as they may reflect secondary or in vitro-specific metabolism.

Some metabolites exhibited unique dynamics. For example, M11 gradually declined early in the experiment, reaching its lowest value between 8 and 12 h, and then recovering significantly at 24 h. M19 gradually declined throughout the incubation period. M14 and M15 kept fluctuating, remaining at moderate-to-low levels overall. In summary, M13 and M3 were the most predominant metabolites in terms of concentration, while M12 can be considered a representative metabolite that dissipates rapidly. M11, on the other hand, represents a metabolite with more unique changes.

#### 2.2.2. Correlation-Based Research of 5F-ADB-PINACA Metabolites

**Figure 4:**
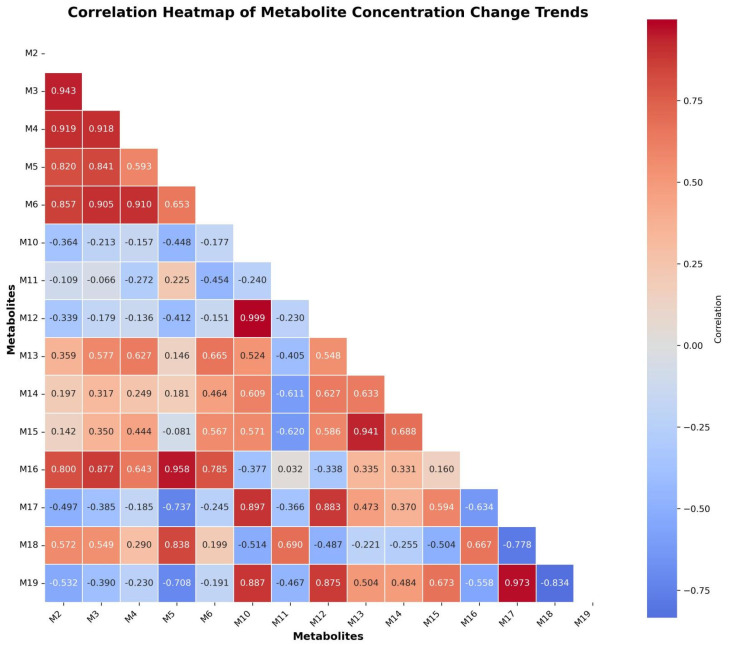
The heatmap of the concentrations over time of 15 selected metabolites of 5F-ADB-PINACA.

In this study, based on a heatmap analysis of the temporal correlation of metabolite concentrations (correlation analyses were based on data obtained from replicate experiments performed over three days), the metabolites of 5F-ADB-PINACA can be broadly divided into four categories (the heatmap of the temporal correlation of metabolite concentrations is shown in Figure 4). It should be emphasized that the correlation analysis provides descriptive insight into co-occurring temporal trends, but it does not establish direct precursor relationships or shared pathways. Accordingly, correlation-based clusters should be viewed as indicative rather than mechanistic. First, the highly correlated cluster (M2, M3, M4, M5, M6, and M7) exhibited highly consistent dynamic changes during incubation, with correlation coefficients generally greater than 0.9, suggesting that these metabolites may be regulated by similar enzymatic reactions. This cluster of metabolites showed an overall increasing trend, representing the primary metabolic transformation.

Second, the dominant stable metabolite (M13) maintained the highest level at all time points and maintained strong correlations with most members of the highly correlated cluster. This stability, coupled with its highest concentration, suggests that M13 is a core metabolite of 5F-ADB-PINACA and may be the most representative metabolite in toxicological testing.

The third category is the fluctuating metabolite. Unlike the positive correlations with other metabolites, the correlations between M11, M14, M15, M16, M18, and M20 were more dispersed, with relatively complex trends. M11, the most concentrated metabolite among these six metabolites, showed a primarily negative correlation. Its level decreased early in the study and then increased back significantly after 24 h, suggesting that its metabolism may involve different metabolic pathways or secondary metabolic mechanisms. M11 represents a specific metabolite with continuously fluctuating levels and, therefore, has a certain value in poisoning detection and toxicological interpretation.

The fourth category consists of rapidly decaying metabolites (M10, M12, M17, and M19). These metabolites reached high levels early in the study and then declined rapidly. M12 had the highest position around 2 h before rapidly decaying, which is typical of short-lived metabolites. Although its detection window is limited and early, it is an important indicator for dynamic tracking of metabolic processes and early detection.

It should be noted that metabolites showing high abundance in the pHLM system may not necessarily represent the most detectable or stable biomarkers in authentic human samples, where renal excretion plays an important role.

Combining the temporal trends of metabolites with the correlation clustering results, M13, M3, M11, and M12 can be identified as potential temporal metabolic signatures of 5F-ADB-PINACA. M13 and M3 represent the main stable product and the rising cluster, respectively. M11 exhibits a unique fluctuation pattern, while M12 can be regarded as a typical representative of a rapidly decaying metabolite.

### 2.3. Identification of 5F-ADBICA Metabolites in HLM Incubation

**Figure 5:**
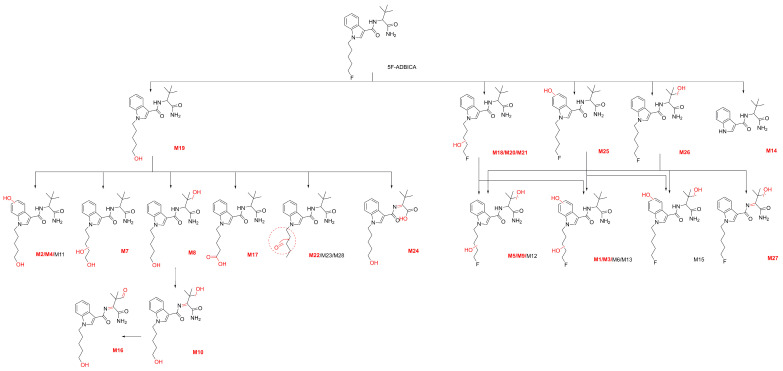
The 28 metabolites and predicted metabolic ways of 5F-ADBICA. (The metabolites highlighted in red were detected in zebrafish).

A total of 28 metabolites of 5F-ADBICA were discovered in the HLM metabolic study. In this experiment, nine metabolic reactions were discovered, including hydroxylation; dihydroxylation; dehydrogenation; amide hydrolysis; hydrolytic defluorination; oxidative to aldehyde; oxidative defluorination to aldehyde; defluorination to carboxylic acid; and N-dealkylation. The LC–MS data of the 28 metabolites, metabolic reactions, and reaction sites of each metabolite are shown in Table 2. All 28 structures of the metabolites and predicted metabolic processes are shown in Figure 5. The following 28 metabolites were analyzed based on their different metabolic reactions. Same as the analysis of the 5F-ADBICA metabolites, we group them according to all the metabolic reactions that occurred with each metabolite.

#### 2.3.1. Hydroxylation

In the metabolism study of 5F-ADBICA, a total of five metabolites underwent hydroxylation reactions. However, there were three metabolic sites, resulting in different product ions. The metabolic sites of M18, M20, and M21 ([M+H]^+^ *m*/*z* 378.2188, Figures S19–S21) were on the 5-fluorine 5-fluoropentyl side chain; the product ions *m*/*z* 144.0444 and 248.1081 indicated the metabolic site (for the fragmentation pathway of M20, see Figure S22). M25 ([M+H]^+^ *m*/*z* 378.2188; Figure S23) was identified as an indole core hydroxylated metabolite, as evidenced by the product ions at *m*/*z* 160.0393 and 248.1081, and the product ions *m*/*z* 144.0444, 232.1132, and 361.1922 showed M26 ([M+H]^+^ *m*/*z* 378.2188, Figure S24) hydroxylated on the ADB residue (for the fragmentation pathway of M26, see Figure S25).

#### 2.3.2. Dihydroxylation

The amount of metabolites with dihydroxylation was the highest. For all metabolites with dihydroxylation, hydroxylations occurred on different parts of the structure. Among these metabolites, the product ions at *m*/*z* 144.0444 and 248.1081 indicated that one hydroxylation of M5, M9, and M12 ([M+H]^+^ *m*/*z* 394.2137) occurred on the 5-fluoropentyl side chain, while the *m*/*z* 377.1871 suggested that the ADB residue was also hydroxylated. For M1, M3, M6, and M13 ([M+H]^+^ *m*/*z* 394.2137), the product ion at *m*/*z* 160.0393 indicated hydroxylation of the indole core, and the ion at *m*/*z* 264.1031 showed an additional hydroxylation on the 5-fluoropentyl side chain. In contrast, M15 ([M+H]^+^ *m*/*z* 394.2137) showed different metabolic sites, with the product ion at *m*/*z* 160.0393 indicating hydroxylation of the indole core and the ions at *m*/*z* 248.1081 and 377.1871 indicating another metabolic site on the ADB residue.

#### 2.3.3. Hydroxylation + Dehydrogenation

M27 ([M+H]^+^ *m*/*z* 376.2031) was identified as the metabolite with hydroxylation and dehydrogenation. The product ions at *m*/*z* 144.0444 and 232.1132 indicated that neither the indole core nor the 5-fluoropentyl side chain was involved in the metabolism; however, the [M+H]^+^ ion demonstrated that hydroxylation and dehydrogenation occurred on the ADB residue (Figure S26).

#### 2.3.4. N-Dealkylation

M14 ([M+H]^+^ *m*/*z* 274.1550), was identified as the metabolite with N-dealkylation. The product ions *m*/*z* 144.0444 and 229.1335 indicated that the whole 5-fluoropentyl side chain was eliminated. Furthermore, fragment ion *m*/*z* 257.1285 also showed that the whole 5-fluoropentyl side chain was eliminated (Figure S27).

#### 2.3.5. Hydrolytic Defluorination

**Figure 6:**
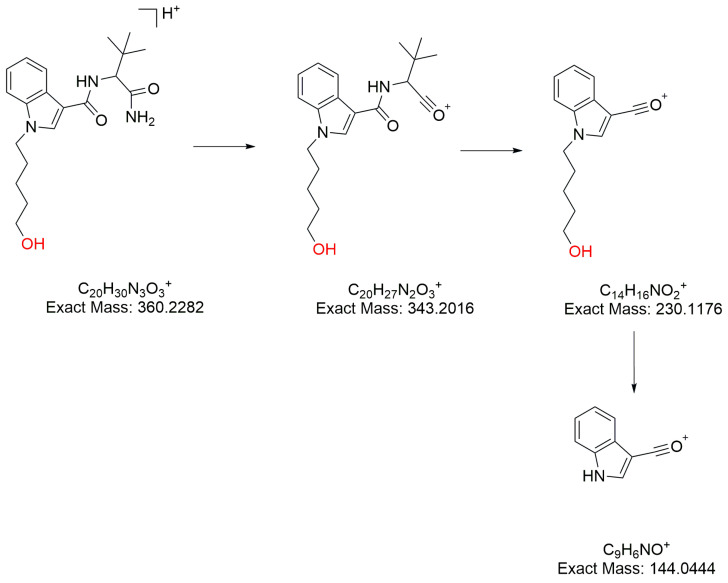
The fragment pathways of M19 of 5F-ADBICA.

M19 ([M+H]^+^ *m*/*z* 360.2282, Figure S28) was identified as the metabolite with hydrolytic defluorination. The [M+H]^+^ and product ions *m*/*z* 144.0444 and 230.1176 indicated the fluorine on the 5-fluoropentyl side chain was hydrolytic defluorination (for the fragmentation pathway of M19, see Figure 6).

#### 2.3.6. Hydrolytic Defluorination + Hydroxylation

A total of five metabolites with hydrolytic defluorination + hydroxylation were identified. However, there were three hydroxylation reaction sites of these five metabolites. For M2, M4, and M11 ([M+H]^+^ *m*/*z* 376.2231), the product ions *m*/*z* 160.0393 and 246.1125 showed that they were hydroxylated on the indole core (Figure S29). Conversely, the product ions *m*/*z* 144.0444 and 246.1125 indicated that M7 ([M+H]^+^ *m*/*z* 376.2231) was hydroxylated on the 5-fluoropentyl side chain, but in M8 ([M+H]^+^ *m*/*z* 376.2231), the product ions *m*/*z* 144.0444 and 230.1176 indicated there was no hydroxylation on the indole core and 5-fluoropentyl side chain; the hydroxylation occurred on the ADB residue (Figure S30).

#### 2.3.7. Defluorination to Aldehyde

A total of three metabolites, M22, M23, and M28 ([M+H]^+^ *m*/*z* 358.2125), were identified as metabolites with defluorination to aldehyde. The product ions *m*/*z* 144.0444 and 228.1019 indicated that the defluorination to aldehyde occurred on the 5-fluoropentyl side chain. Based on previous studies, both terminal and internal ketones were inferred to be present, possibly resulting from further oxidation of the terminal hydroxyl group formed via defluorination (Figure S31).

#### 2.3.8. Hydrolytic Defluorination + Dehydrogenation + Hydroxylation

M10 ([M+H]^+^ *m*/*z* 374.2074) was identified as the metabolite with hydrolytic defluorination, dehydrogenation, and hydroxylation. The product ions *m*/*z* 144.0444 and 230.1176 indicated that the hydrolytic defluorination was on the 5-fluoropentyl side chain. In addition, the [M+H]^+^ ion demonstrated that hydroxylation and dehydrogenation took place on the ADB residue (Figure S32).

#### 2.3.9. Hydrolytic Defluorination + Dehydrogenation + Amide Hydrolysis

M24 ([M+H]^+^ *m*/*z* 359.1965) was the metabolite with hydrolytic defluorination + dehydrogenation + amide hydrolysis. The product ions *m*/*z* 144.0444 and 230.1176 showed that the hydrolytic defluorination occurred on the fluorine. Furthermore, the [M+H]^+^ ion of M24 suggested that dehydrogenation and amide hydrolysis took place on the ADB residue (Figure S33).

#### 2.3.10. Hydrolytic Defluorination + Dehydrogenation + Oxidation to Aldehyde

M16 ([M+H]^+^ *m*/*z* 372.1918) was identified as a metabolite with hydrolytic defluorination, dehydrogenation, and oxidation to aldehyde. The product ions *m*/*z* 144.0444 and 230.1176 showed that the 5-fluoropentyl side chain has been hydrolyzed. Similar to M10 of 5F-ADB-PINACA, the degree of unsaturation of the ADB residue in M16 increased by two. Therefore, an aldehyde group was inferred to be formed on the butyl moiety of the ADB residue, accompanied by dehydrogenation of the ADB group (Figure S34).

#### 2.3.11. Defluorination to Carboxylic Acid

M17 ([M+H]^+^ *m*/*z* 374.2074) stands as the sole metabolite undergoing carboxylation. The product ions *m*/*z* 101.0597, 144.0444, and 244.0968 showed the defluorination to carboxylic acid (Figure S35).

### 2.4. Temporal Metabolic Profiling and Potential Metabolite Biomarker of 5F-ADBICA

#### 2.4.1. Temporal Trends of 5F-ADBICA Metabolites

**Figure 7:**
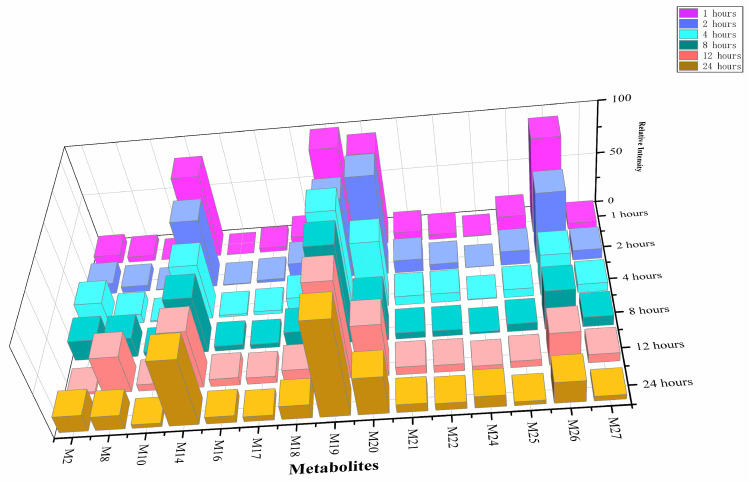
The relative intensity of changes over time of 15 selected metabolites of 5F-ADBICA.

After 24 h of incubation, the parent drug 5F-ADBICA was completely metabolized. We selected the 15 metabolites with the highest relative concentrations and present them in Figure 7, which shows the change in relative intensities over time. M19 maintained the highest level and was the most abundant metabolite. The intensity of M8 continued to rise early in the experiment, reaching a peak around 12 h. M24 and M16 also showed an upward trend but to a lesser extent. Meanwhile, M26, M20, and M25 gradually declined over time, with M26 being the most representative, reaching a peak early before rapidly declining. M14, M17, M18, M21, and M27 were relatively stable, but their overall level was low.

Furthermore, M19 maintained high levels throughout the experiment and continued to be the most abundant. M8 peaked at 12 h and then declined significantly at 24 h. At last, M19 was the main metabolite in terms of concentration, while M26 can be considered a representative metabolite that declined rapidly.

#### 2.4.2. Correlation-Based Research of 5F-ADBICA Metabolites

**Figure 8:**
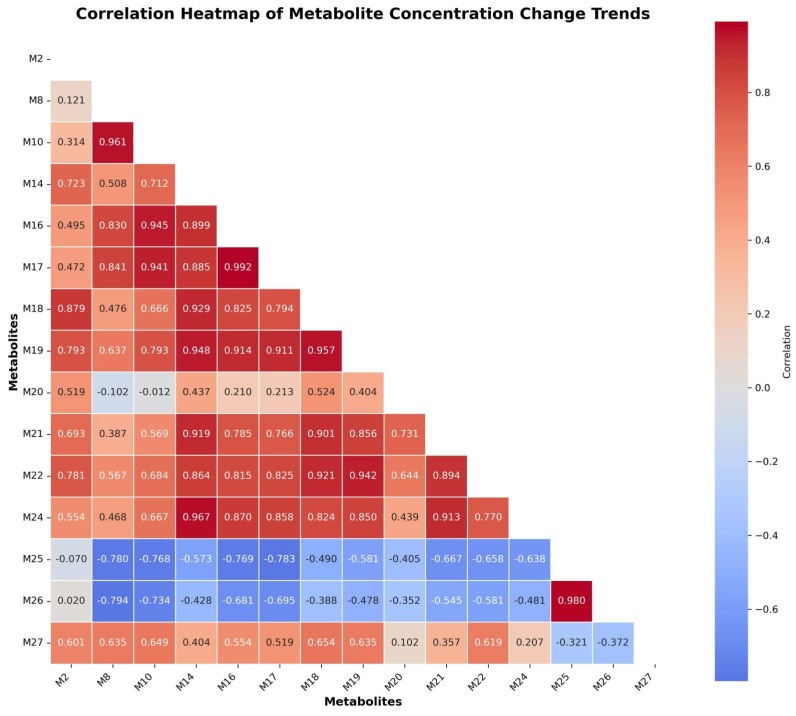
The heatmap of concentrations over time of 15 selected metabolites of 5F-ADBICA.

Correlation heatmap analysis revealed that 5F-ADBICA metabolites were broadly classified into four categories. Correlation analyses were based on data obtained from replicate experiments performed over three days (the heatmap of the temporal correlation of metabolite concentrations is shown in Figure 8).

First, the core strongly correlated cluster (M10, M14, M16, M17, M18, M19, M21, M22, and M24) exhibited strong consistency, with correlation coefficients generally >0.8, constituting the primary metabolic network. M19, M16, and M17 were highly connected metabolites.

Second, the strongly negatively correlated pairs (M25 and M26) exhibited correlations close to 1.0 but exhibited broad negative correlations with the core cluster (ρ values ranging from −0.6 to −0.9), suggesting metabolic processes that diverge from the main pathway and are likely driven by distinct enzyme systems.

Third, the transitional metabolites (M20 and M27) exhibited low-to-moderate correlations with the core cluster and relatively independent dynamic patterns, suggesting their potential involvement in metabolic processes under specific conditions. Finally, a small number of metabolites showed moderate correlation with the core cluster, but the overall trend was weak, indicating that they were not too important.

In summary, M19, M16, and M14 can be regarded as stable representatives of the core metabolic cluster, whereas M25 and M26 exhibit pronounced reverse metabolic characteristics. Meanwhile, M20 and M27 are indicative of branch-type metabolic pathways.

Combining the temporal trends and correlation clustering results of metabolites, M19, M20, and M26 can be hypothesized to be potential temporal metabolic signatures of 5F-ADBICA. M19 represents the major stable product and an increasing cluster. M20 represents an independent metabolic trend, while M26 can be considered a typical representative of a rapidly decaying metabolite.

### 2.5. Metabolites Detection in Zebrafish Studies

In two in vivo zebrafish experiments conducted on two SCs, we detected 16 5F-ADB-PINACA phase I metabolites and 20 5F-ADBICA phase I metabolites after a 6 h incubation period. When the experiment was extended to 12 h, we detected 19 5F-ADB-PINACA phase I metabolites and 21 5F-ADBICA phase I metabolites. All metabolites detected in the zebrafish experiments are highlighted in Figure 1 and Figure 5. Compared to the 6 h results, new metabolites emerged for both parent drugs. These metabolites were also detected in HLM. All potential temporal metabolic signatures of 5F-ADB-PINACA (M3, M11, M12, and M13) identified through HLM and temporal metabolic profiling were also detected in the zebrafish metabolism studies. Similarly, all three potential temporal metabolic signatures of 5F-ADBICA (M19, M20, and M26) were also detected in the zebrafish studies. These metabolites detected in the zebrafish experiments validated the results of the pHLM study.

The differences in metabolites observed between human liver microsomes (HLM) and zebrafish can be attributed to several methodological and biological factors. First, the concentrations of compounds used in the two models are not the same, which affects enzyme saturation and the abundance of metabolites. Second, zebrafish possess a different range of CYP450 isoenzymes than human liver microsomes, which may lead to different metabolic pathways. Finally, in the experiments, zebrafish were administered drugs via continuous ingestion of aqueous solutions, while liver microsomes were administered drugs in a single dose, resulting in different drug absorption pathways. These factors combined may explain the differences in metabolite intensity patterns between the two models.

In addition to general differences in CYP450 composition, previous studies have shown that zebrafish express functionally conserved but quantitatively distinct CYP isoforms compared with humans, which may preferentially favor certain oxidative or defluorination pathways. Furthermore, continuous aqueous exposure in zebrafish may promote prolonged biotransformation, whereas pHLM incubation may emphasize rapid metabolism. These factors together could contribute to the observed differences in metabolite profiles [14,15,16,17].

### 2.6. Comparison of Metabolites Between 5F-ADB-PINACA and 5F-ADBICA

Clearly, due to the differences in their core structures, the results indicate that the two synthetic cannabinoids still exhibit certain differences in their metabolic processes, alhough the mainly metabolic reactions are generally consistent (hydroxylation; dihydroxylation; dehydrogenation; amide hydrolysis; hydrolytic defluorination; and defluorination to carboxylic acid). In addition, it can be found that some metabolites of the two SCs only differ in their core structures, but the metabolic reactions were the same. However, a very small number of metabolite structures and metabolite reactions are unique to one drug. However, the two most important metabolite biomarkers of 5F-ADB-PINACA and 5F-ADBICA are structurally similar compounds: M13 in 5F-ADB-PINACA and M19 in 5F-ADBICA. Both undergo hydrolytic defluorination. Combined with correlation analysis, we found that M13 of 5F-ADB-PINACA and M19 of 5F-ADBICA represent their respective main metabolic core strongly correlated cluster with high correlation, indicating that the overall metabolic reaction trends of the two substances are similar. At the same time, M12 of 5F-ADB-PINACA and M20 of 5F-ADBICA both underwent hydroxylation reactions on the5-fluoropentyl side chain and also had similar structures. However, considering the differences in several other temporal metabolic signatures and the different metabolic reactions of some other metabolites, it is clear that some metabolic changes occurred due to changes in the core structure, such as the defluorination to aldehyde on the 5F-ADBICA.

A comparison of 5F-ADB-PINACA and 5F-ADBICA reveals a significantly faster decline of 5F-ADB-PINACA. While both exhibit rapid metabolic speed, the overall trend suggests that 5F-ADB-PINACA exhibits less metabolic stability and disappears more rapidly. This may be due to the fact that 5F-ADB-PINACA primarily completes its metabolic reactions rapidly through a few efficient metabolic pathways, limiting its metabolite diversity. 5F-ADBICA maintains a consistent concentration for a longer period, allowing it to be continuously processed by multiple enzymes, resulting in a greater diversity of metabolites with potentially different pharmacological relevance. However, no pharmacological or receptor binding data were generated in this study, and therefore, no conclusions regarding metabolite potency can be drawn.

Combining metabolite intensities, 5F-ADBICA exhibits a higher number of metabolites, suggesting that its core structure may be more reacted by metabolic enzymes, leading to the production of a diverse range of metabolites. Therefore, the differences between 5F-ADB-PINACA and 5F-ADBICA are reflected not only in their disappearance rates but also in their metabolite production patterns, indicating that the core structure plays a key role in metabolic processes.

One limitation of this study is the lack of real human data. While human liver microsomes (HLM) and zebrafish are valuable models for early metabolic characterization, their metabolic outcomes still differ slightly from those in humans. HLMs cannot reflect extrahepatic metabolism or inter-individual variability, and zebrafish CYP isoenzymes only partially overlap with the human CYP450 family. Therefore, the metabolic findings identified in this study should be considered predictive rather than definitive. Future studies using real human biological samples are crucial for validating the proposed metabolic pathways and confirming the applicability of potential metabolite biomarkers in forensic and clinical toxicology.

## 3. Materials and Methods

### 3.1. Chemicals and Reagents

Liquid chromatography–mass spectrometry (LC–MS)-grade acetonitrile (ACN) was purchased from Merck (Darmstadt, Germany). Formic acid (FA) was purchased from Thermo Fisher Scientific (San Jose, CA, USA). Ultrapure water was purified using a Millipore Milli-Q water purification system (Millipore, Bedford, MA, USA). 5F-ADB-PINACA and 5F-ADBICA solid reference standards were obtained from the National Narcotics Laboratory (Ministry of Public Security, Beijing, China). Pooled human liver microsomes (pHLM, protein concentration 20 mg/mL) were purchased from Beijing iphase Biotechnology Co., Ltd. (Beijing, China). NADPH regeneration system solutions A and B were purchased from Beijing iphase Biotechnology Co., Ltd. (Beijing, China). Phosphate-buffered saline (PBS, 0.1 mol/L) was purchased from Beijing iphase Biotechnology Co., Ltd. (Beijing, China). Adult zebrafish were obtained from the School of Forensic Medicine, Shanxi Medical University (Taiyuan, China).

### 3.2. UPLC/QE Orbitrap Mass Spectrometry Conditions

Samples were separated using a Waters BEH C18 column (100 × 2.5 mm, 1.7 μm; Waters, Milford, MA, USA) and an Ultimate Ultra-High-Performance Liquid Chromatography (UHPLC) system (Thermo Fisher Scientific, San Jose, CA, USA). The column temperature was maintained at 40 °C, the flow rate was set at 0.3 mL/min, and the autoinjection volume was set to 5 μL. Mobile phases A and B were 0.1% FA in water and 0.1% FA in acetonitrile, respectively. The 20 min elution program was as follows: 5% B (0–0.5 min); 5% to 95% B (0.5–12 min); hold B at 95% (12–18 min); 95% to 5% B (18–18.1 min); hold B at 5% (18.1–20 min). Mass spectrometric analysis was performed using a Q Exactive PLUS Orbitrap MS (Thermo Fisher Scientific, San Jose, CA, USA) equipped with an electrospray ionization (ESI) source set in positive mode. The ionization source was setting as following parameters: spray voltage, 3.50 kV; normalized collision energy (NCE), 20 eV, 40 eV, 60 eV; ion transfer capillary temperature, 320 °C; auxiliary gas heating temperature, 320 °C; sheath gas (N2) flow rate, 35 arbitrary units (AUs); auxiliary (N2) gas flow rate, 10 AUs; sweep gas (N2), 0 AUs. The conditions of the precursor ion full-scans (MS1) were set as follows: scan range 200 to 600 *m*/*z*; resolution, 70,000, automatic gain control (AGC) target, 5.0 × 10^5^; maximum IT, 100 ms. The parameters of dd-MS2 discovery were set as follows: NCE, 20, 40, and 60; resolution, 17,500; AGC target, 5.0 × 10^4^; maximum IT, 100 ms; isolation window, 1.0 *m*/*z*.

### 3.3. Human Liver Microsome Incubation Method

SCs 5F-ADB-PINACA and 5F-ADBICA were prepared in acetonitrile at a concentration of 1 mg/mL. The pHLM incubation system for both 5F-ADB-PINACA and 5F-ADBICA consisted of 10 μL of the target drug (1 mg/mL) in acetonitrile, 50 μL of pHLM, 50 μL of NADPH regeneration system solution A, 10 μL of NADPH regeneration system solution B, and 880 μL of PBS (0.1 mol/L), for a total volume of 1 mL. In the experiment, the incubation system without the target drug was preincubated at 37 °C for 5 min before the target drug was added. After the target drug was added, the incubation system was incubated at 37 °C for 24 h. A total of 100 μL of samples was removed from the system at 1, 2, 4, 8, 12, and 24 h. The reaction was terminated by adding 100 μL of acetonitrile to the removed samples. The samples were then centrifuged at 13,000× *g* for 10 min at 4 °C. A total of 100 μL of the supernatant was transferred to an injection vial. The blank system consisted of three groups: no target drug, no NADPH solution, and no human liver microsomes or NADPH solution.

### 3.4. Zebrafish Method

SCs 5F-ADB-PINACA and 5F-ADBICA were prepared in aqueous solutions at 1 μg/mL. Adult male and female zebrafish (0.8–1.2 g) were randomly divided into three groups of six fish each. Two groups were treated with aqueous solutions containing 5F-ADB-PINACA and 5F-ADBICA (1 μg/mL), respectively, while the last group remained in pure water. After the addition of the solutions at 6 and 12 h, three zebrafishes were removed from each group, washed with pure water, sacrificed, and ground homogenously using a tissue grinder. A total of 2 mL of acetonitrile was added, shaken thoroughly, and 500 μL of the mixture was collected. A total of 500 μL of acetonitrile was added to the homogenate, and the mixture was centrifuged at 15,000× *g* for 10 min at 4 °C. A total of 200 μL of the supernatant was transferred to a sample vial.

### 3.5. Data Analysis Method

For data analysis, accurate masses for all compounds in this study were calculated using Mass Frontier 8.0 software (Thermo Fisher Scientific, USA), and metabolite structures were determined using Compound Discover 3.2 software (Thermo Fisher Scientific, USA). Metabolite identification was based on precise mass measurements (±5 ppm), MS/MS fragment information, elemental composition prediction, and known biotransformation patterns. The data of temporal trends of metabolites were normalized to the most abundant. For correlation analysis, Pearson correlation analysis was performed on metabolite data at different incubation time points using self written Python Version 3.9.13 code, and metabolites with |ρ| > 0.8 were classified into strongly correlated clusters while minimizing noise from minor fluctuations. Replicated data from three independent experiments were mean sampled before correlation analysis to ensure comparability.

## 4. Conclusions

This study investigated the metabolism of 5F-ADB-PINACA and 5F-ADBICA in SCs using a pHLM culture system and zebrafish. Metabolite identification was performed using a UHPLC-QE Obitrap MS system. A total of 21 metabolites of 5F-ADB-PINACA and 28 metabolites of 5F-ADBICA were detected. The main metabolic reactions involved in this study included hydroxylation; dihydroxylation; dehydrogenation; amide hydrolysis; hydrolytic defluorination; and defluorination to carboxylic acid. Temporal metabolic profiling was conducted by combining temporal trends of metabolites and correlation-based research. The metabolites derived from the two SCs were categorized according to their distinct metabolic patterns. Temporal trends combined with correlation-based analyses enabled a classification of these metabolites, through which potential metabolic screening markers were identified.

The comprehensive study results showed that metabolite M13 (hydrolytic defluorination) can serve as a potential metabolite biomarker for 5F-ADB-PINACA. M19 (hydrolytic defluorination) is a potential metabolite biomarker for 5F-ADBICA. Meanwhile, these two potential metabolite biomarkers selected are unique to these two SCs. The results indicate that these two synthetic cannabinoids, which differ only in their core structure, have potential metabolite biomarkers produced by the same metabolic reaction. The potential metabolite biomarkers obtained in this study can provide a basis for the identification of such SCs in biological samples and provide a reference for the study of the metabolic mechanisms of other novel SCs.

## Figures and Tables

**Table 1:** Metabolites of 5F-ADB-PINACA detected from pHLM incubation and the corresponding metabolic reactions of each metabolite, with accurate [M+H]^+^ UHPLC-MS data (retention times, accurate product ions, and mass error).

Chemical Structure of 5F-ADB-PINACA	Metabolites ID	Metabolic Reaction			Retention Time (min)	Chemical Formula	Accurate [M+H]^+^ (*m*/*z*)	Mass Error (ppm)	Accurate Product Ions (*m*/*z*)
		ADB	IND	5FB					
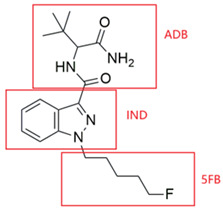	5F-ADB- PINACA				8.21	C_19_H_26_FN_4_O_2_	363.2191	1.38	145.0396, 233.1085, 251.1190, 318.1976
	M1			+2OH	5.56	C_19_H_27_FN_4_O_4_	395.2089	0.76	145.0396, 163.0502, 267.1140, 378.1824
	M2			F → OH, +OH	5.62	C_19_H_28_N_4_O_4_	377.2183	0.80	145.0396, 229.0972, 247.1077, 332.1969, 360.1918
	M3	+OH		F → OH	5.67	C_19_H_28_N_4_O_4_	377.2183	1.06	145.0396, 213.1022, 231.1128, 360.1918
	M4			F → OH, +OH	5.72	C_19_H_28_N_4_O_4_	377.2183	1.06	145.0396, 229.0972, 247.1077, 332.1969, 360.1918
	M5	+OH, Dehydrogenation		F → OH	5.77	C_19_H_26_N_4_O_4_	375.2027	0.53	145.0396, 213.1022, 231.1128, 357.1921
	M6		+OH	F → OH	6.05	C_19_H_28_N_4_O_4_	377.2183	1.06	161.0346, 179.0451, 229.0972, 247.1077, 332.1969
	M7	Dehydrogenation		F → OH, +OH	6.08	C_19_H_26_N_4_O_4_	375.2027	0.53	145.0396, 229.0972, 245.0921, 330.1812, 358.1761
	M8	+OH		F → Aldehyde	6.30	C_19_H_26_N_4_O_4_	375.2027	0.53	145.0396, 163.0502, 201.1022, 330.1812, 358.1761
	M9	Oxidation → Aldehyde Dehydrogenation		F → OH	6.38	C_19_H_24_N_4_O_4_	373.1870	0.80	145.0396, 163.0502, 213.1022, 231.1128
	M10			+OH	6.57	C_19_H_27_FN_4_O_3_	379.2140	0.79	145.0396, 231.0928, 249.1034, 267.1140, 334.1925
	M11			F → COOH	6.59	C_19_H_26_N_4_O_4_	375.2027	0.53	145.0396, 217.0972, 245.0921, 330.1812, 358.1761
	M12			+OH	6.64	C_19_H_27_FN_4_O_3_	379.2140	0.79	145.0396, 231.0928, 249.1034, 267.1140, 334.1925
	M13			F → OH	6.66	C_19_H_28_N_4_O_3_	361.2234	0.83	145.0396, 213.1022, 231.1128, 316.2020, 344.1969
	M14			+OH	6.69	C_19_H_27_FN_4_O_3_	379.2140	0.79	145.0396, 231.0928, 249.1034, 267.1140, 334.1925
	M15			+OH	6.90	C_19_H_27_FN_4_O_3_	379.2140	0.79	145.0396, 231.0928, 249.1034, 267.1140, 334.1925
	M16	NH_2_ → OH Dehydrogenation		F → OH	7.08	C_19_H_25_N_3_O_4_	360.1918	0.56	145.0396, 163.0502, 213.1021, 231.1128, 360.1918
	M17			+OH	7.12	C_19_H_27_FN_4_O_3_	379.2140	0.79	145.0396, 233.1085, 251.1190, 362.1874
	M18	NH_2_ → OH		F → OH	7.29	C_19_H_27_N_3_O_4_	362.2074	0.55	145.0396, 213.1022, 231.1128, 316.2020
	M19		+OH		7.42	C_19_H_27_FN_4_O_3_	379.2140	0.79	161.0346, 179.0451, 249.1034, 267.1140, 334.1925
	M20	NH_2_ → OH		+OH	7.54	C_19_H_26_FN_3_O_4_	380.1980	0.79	145.0396, 251.1190, 304.1820, 362.1874
	M21	Deamination	+OH	+OH	8.04	C_19_H_26_FN_3_O_4_	380.1980	0.79	161.0346, 179.0451, 229.0972, 267.1140, 334.1925

NH_2_ → OH: amide hydrolysis; +OH: hydroxylation; F → OH: defluorination to alcohol; F → COOH: defluorination to carboxylic acid; F → Aldehyde: defluorination to aldehyde; +2OH: Dihydroxylation.

**Table 2:** Metabolites of 5F-ADBICA detected from pHLM incubation and the corresponding metabolic reactions of each metabolite, with accurate [M+H]^+^ UHPLC-MS data (retention times, accurate product ions, and mass error).

Chemical Structure of 5F-ADBICA	Metabolites ID	Metabolic Reaction			Retention Time (min)	Chemical Formula	Accurate [M+H]^+^ (*m*/*z*)	Mass Error (ppm)	Accurate Product Ions (*m*/*z*)
		ADB	IND	5FB					
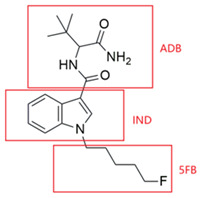	5F-ADBICA				7.95	C_20_H_28_FN_3_O_2_	362.2238	0.00	144.0444, 232.1132, 345.1973
	M1		+OH	+OH	5.37	C_20_H_27_FN_3_O_4_	394.2137	1.27	160.0393, 264.1031, 377.1871
	M2		+OH	F → OH	5.39	C_20_H_29_N_3_O_4_	376.2231	1.33	160.0393, 246.1125
	M3		+OH	+OH	5.49	C_20_H_27_FN_3_O_4_	394.2137	1.27	160.0393, 264.1031, 377.1871
	M4		+OH	F → OH	5.52	C_20_H_29_N_3_O_4_	376.2231	1.33	160.0393, 246.1125, 359.1965
	M5	+OH		+OH	5.53	C_20_H_27_FN_3_O_4_	394.2137	1.27	144.0444, 248.1081, 377.1871
	M6		+OH	+OH	5.6	C_20_H_27_FN_3_O_4_	394.2137	1.27	160.0393, 264.1031, 377.1871
	M7			F → OH, +OH	5.61	C_20_H_29_N_3_O_4_	376.2231	1.33	144.0444, 246.1125, 359.1965
	M8	+OH		F → OH	5.66	C_20_H_29_N_3_O_4_	376.2231	1.33	144.0444, 230.1176, 359.1965
	M9	+OH		+OH	5.69	C_20_H_27_FN_3_O_4_	394.2137	1.27	144.0444, 248.1081, 377.1871
	M10	Dehydrogenation, +OH		F → OH	5.72	C_20_H_27_N_3_O_4_	374.2074	1.34	144.0444, 230.1176
	M11		+OH	F → OH	5.77	C_20_H_29_N_3_O_4_	376.2231	1.33	160.0393, 246.1125, 359.1965
	M12	+OH		+OH	5.83	C_20_H_27_FN_3_O_4_	394.2137	1.27	144.0444, 248.1081, 377.1871
	M13		+OH	+OH	5.85	C_20_H_27_FN_3_O_4_	394.2137	1.27	160.0393, 264.1031, 377.1871
	M14			N-dealkylation	5.85	C_15_H_19_N_3_O_2_	274.1550	0.73	144.0444, 229.1335, 257.1285
	M15	+OH	+OH		6.02	C_20_H_27_FN_3_O_4_	394.2137	1.27	160.0393, 248.1031, 377.1871
	M16	Dehydrogenation, Oxidation → Aldehyde		F → OH	6.20	C_20_H_25_N_3_O_4_	372.1918	1.61	144.0444, 230.1176
	M17			F → COOH	6.47	C_20_H_26_N_3_O_4_	374.2074	1.34	101.0597, 144.0444, 244.0968, 357.1809
	M18			+OH	6.47	C_20_H_28_FN_3_O_3_	378.2188	0.53	144.0444, 248.1081, 361.1922
	M19			F → OH	6.54	C_20_H_29_N_3_O_3_	360.2282	0.83	144.0444, 230.1176, 343.2016
	M20			+OH	6.60	C_20_H_28_FN_3_O_3_	378.2188	0.53	144.0444, 248.1081, 361.1922
	M21			+OH	6.72	C_20_H_28_FN_3_O_3_	378.2188	0.53	144.0444, 248.1081, 361.1922
	M22			F → Aldehyde	6.72	C_20_H_27_N_3_O_3_	358.2125	1.40	144.0444, 228.1019, 341.1860
	M23			F → Aldehyde	6.84	C_20_H_27_N_3_O_3_	358.2125	1.40	144.0444, 228.1019
	M24	NH2 → OH, Dehydrogenation		F → OH	6.86	C_20_H_26_N_2_O_4_	359.1965	0.84	144.0444, 230.1176
	M25		+OH		7.01	C_20_H_28_FN_3_O_3_	378.2188	0.53	160.0393, 248.1081, 361.1922
	M26	+OH			7.05	C_20_H_28_FN_3_O_3_	378.2188	0.53	144.0444, 232.1132, 361.1922
	M27	+OH, Dehydrogenation			7.07	C_20_H_26_FN_3_O_3_	376.2031	1.33	144.0444, 232.1132
	M28			F → Aldehyde	7.16	C_20_H_27_N_3_O_3_	358.2125	1.40	144.0444, 228.1019, 341.1860

NH_2_ → OH: amide hydrolysis; +OH: hydroxylation; F → OH: defluorination to alcohol; F → COOH: defluorination to carboxylic acid; +2OH: dihydroxylation; F → aldehyde: oxidation to aldehyde.

## Data Availability

Data are contained within the article and Supplementary Materials.

## Supplementary Materials

The following supporting information can be downloaded at: https://www.mdpi.com/article/10.3390/molecules31020250/s1, Figure S1: The MS2 spectrum of M10; Figure S2: The MS2 spectrum of M12; Figure S3: The MS2 spectrum of M14; Figure S4: The MS2 spectrum of M15; Figure S5: The MS2 spectrum of M17; Figure S6: The fragmentation pathway of M12; Figure S7: The MS2 spectrum of M19; Figure S8: The MS2 spectrum of M13; Figure S9: The MS2 spectrum of M2; Figure S10: The MS2 spectrum of M4; Figure S11: The fragmentation pathway of M3; Figure S12: The MS2 spectrum of M3; Figure S13: The MS2 spectrum of M6; Figure S14: The MS2 spectrum of M18; Figure S15: The MS2 spectrum of M5; Figure S16: The MS2 spectrum of M16; Figure S17: The MS2 spectrum of M11; Figure S18: The fragmentation pathway of M11; Figure S19: The MS2 spectrum of M18; Figure S20: The MS2 spectrum of M20; Figure S21: The MS2 spectrum of M21; Figure S22: The fragmentation pathway of M20; Figure S23: The MS2 spectrum of M25; Figure S24: The MS2 spectrum of M26; Figure S25: The fragmentation pathway of M26; Figure S26: The MS2 spectrum of M27; Figure S27: The MS2 spectrum of M14; Figure S28: The MS2 spectrum of M19; Figure S29: The MS2 spectrum of M2; Figure S30: The MS2 spectrum of M8; Figure S31: The MS2 spectrum of M22; Figure S32: The MS2 spectrum of M10; Figure S33: The MS2 spectrum of M24; Figure S34: The MS2 spectrum of M16; Figure S35: The MS2 spectrum of M17.

## Abbreviations

The following abbreviations are used in this manuscript:

SCs	Synthetic cannabinoids
NPS	New psychoactive substances
CB_1_	Cannabinoid receptor type 1
THC	Δ^9^-tetrahydrocannabinol
HLM	Human liver microsomes
CYP450	Cytochrome P450 enzyme family
UHPLC	Ultra-high-performance liquid chromatography
UHPLC–QE Orbitrap–MS	Ultra-high-performance liquid chromatography–Q Exactive Orbitrap mass spectrometry
LC–MS	Liquid chromatography–mass spectrometry
HRMS	High-resolution mass spectrometry
MS/MS	Tandem mass spectrometry
MS2	Secondary mass spectrometry
MS1	Precursor ion full-scan mass spectrometry
NCE	Normalized collision energy
AGC	Automatic gain control
IT	Injection time
*m*/*z*	Mass-to-charge ratio
ppm	Parts per million
ESI	Electrospray ionization
PBS	Phosphate-buffered saline
FA	Formic acid
ACN	Acetonitrile
NADPH	Nicotinamide adenine dinucleotide phosphate
AUs	Arbitrary units

## References

[1] UNODC First Reported NPS in 2023 Show Strong Increase in Synthetic Opioids as Stimulants and Synthetic Cannabinoids Diversify. https://www.unodc.org/LSS/Announcement/Details/89a02deb-3344-4308-9980-ca4ca4dc8671.

[2] European Union Drugs Agency (2025). European Drug Report 2025: Trends and Developments.

[3] European Union Drugs Agency (2023). European Drug Report 2023: Trends and Developments.

[4] Vaccaro G., Stair J.L., Kirton S.B., Baker D., Guirguis A. (2025). Screening and quantification of the synthetic cannabinoid receptor agonist 5F-MDMB-PINACA from seized prison paper using ultraperformance liquid chromatography-mass spectrometry approaches. Drug Test. Anal..

[5] Giorgetti A., Brunetti P., Pelotti S., Auwärter V. (2022). Detection of AP-237 and synthetic cannabinoids on an infused letter sent to a German prisoner. Drug Test. Anal..

[6] Alzu’bi A., Almahasneh F., Khasawneh R., Abu-El-Rub E., Bani Baker W., Al-Zoubi R.M. (2024). The synthetic cannabinoids menace: A review of health risks and toxicity. Eur. J. Med. Res..

[7] Banister S.D., Moir M., Stuart J., Kevin R.C., Wood K.E., Longworth M., Wilkinson S.M., Beinat C., Buchanan A.S., Glass M. (2015). Pharmacology of indole and indazole synthetic cannabinoid designer drugs AB-FUBINACA, ADB-FUBINACA, AB-PINACA, ADB-PINACA, 5F-AB-PINACA, 5F-ADB-PINACA, ADBICA, and 5F-ADBICA. ACS Chem. Neurosci..

[8] Iwatsubo T., Hirota N., Ooie T., Suzuki H., Shimada N., Chiba K., Ishizaki T., Green C.E., Tyson C.A., Sugiyama Y. (1997). Prediction of in vivo drug metabolism in the human liver from in vitro metabolism data. Pharmacol Ther..

[9] Giorgetti A., Brunetti P., Haschimi B., Busardò F.P., Pelotti S., Auwärter V. (2023). Human phase-I metabolism and prevalence of two synthetic cannabinoids bearing an ethyl ester moiety: 5F-EDMB-PICA and EDMB-PINACA. Drug Test. Anal..

[10] Körmöczi T., Sija É., Institóris L., Kereszty É.M., Ilisz I., Berkecz R. (2021). Analytical Methodologies for the Characterization and Analysis of the Parent Compound and Phase I Metabolites of 4F-MDMB-BICA in Human Microsome, Urine and Blood Samples. J. Anal. Toxicol..

[11] Ning Y., Wang T., Yang X., Guo F., Xu Y., Zhang Y., Wang K., Hu M., Chen Z., Wei Z. (2025). Systematic Characterization of In Vitro and In Vivo Metabolic Pathways and Identification of Novel Biomarkers of 26 Synthetic Cannabinoids. Molecules.

[12] Presley B.C., Castaneto M.S., Logan B.K., Jansen-Varnum S.A. (2020). Metabolic profiling of synthetic cannabinoid 5F-ADB and identification of metabolites in authentic human blood samples via human liver microsome incubation and ultra-high-performance liquid chromatography/high-resolution mass spectrometry. Rapid Commun. Mass Spectrom..

[13] Giorgetti A., Zschiesche A., Groth O., Haschimi B., Scheu M., Pelletti G., Fais P., Musshoff F., Auwärter V. (2024). ADB-HEXINACA—A novel synthetic cannabinoid with a hexyl substituent: Phase I metabolism in authentic urine samples, a case report and prevalence on the German market. Drug Test. Anal..

[14] Loerracher A.K., Braunbeck T. (2021). Cytochrome P450-dependent biotransformation capacities in embryonic, juvenile and adult stages of zebrafish (Danio rerio)-a state-of-the-art review. Arch. Toxicol..

[15] Chaturvedi K., Hewamanna I., Pandey P., Khan W., Wang Y.-H., Chittiboyina A.G., Doerksen R.J., Godfrey M. (2023). Identification of the Putative Binding Site of a Benzimidazole Opioid (Etazene) and Its Metabolites at µ-Opioid Receptor: A Human Liver Microsomal Assay and Systematic Computational Study. Molecules.

[16] Xu L., Liu X., Song Z., Xiang P., Hang T., Yan H. (2024). In vitro and in vivo metabolism of 3-Methoxyeticyclidine in human liver microsomes, a zebrafish model, and two human urine samples based on liquid chromatography-high-resolution mass spectrometry. Drug Test. Anal..

[17] Camuto C., De-Giorgio A., Corli I., Bilel S., Mazzarino M., Marti M., Botrè F. (2025). Metabolic profiling of the synthetic cannabinoid APP-CHMINACA by in vitro and in vivo models. Forensic Toxicol..

[18] Dvorácskó S., Körmöczi T., Sija É., Bende B., Weiczner R., Varga T., Ilisz I., Institóris L., Kereszty É.M., Tömböly C. (2023). Focusing on the 5F-MDMB-PICA, 4F-MDMB-BICA synthetic cannabinoids and their primary metabolites in analytical and pharmacological aspects. Toxicol. Appl. Pharmacol..

[19] Li H., Qian Z., Zhao Y., Zheng H. (2022). Study on the metabolic process of synthetic cannabinoids 4F-MDMB-BINACA and 4F-MDMB-BICA in human liver microsome and zebrafish model via UHPLC-QE Orbitrap MS. Anal. Bioanal. Chem..

[20] Fong C.Y., Moy H.Y. (2024). Detection of ADB-4en-PINACA Metabolite in Authentic Urine Samples. Emerg. Trends Drugs Addict. Health.

[21] Baginski S.R., Feliu-Pascual A.L., Moore K.N., Concheiro M., Kerrigan S. (2023). The metabolic profile of the synthetic cannabinoid receptor agonist ADB-HEXINACA using human hepatocytes, LC-QTOF-MS and synthesized reference standards. J. Anal. Toxicol..

[22] Wohlfarth A., Castaneto M.S., Zhu M., Pang S., Scheidweiler K.B., Kronstrand R., Huestis M.A. (2015). Pentylindole/Pentylindazole Synthetic Cannabinoids and Their 5-Fluoro Analogs Produce Different Primary Metabolites: Metabolite Profiling for AB-PINACA and 5F-AB-PINACA. AAPS J..

[23] Zhou F., Wang X., Tan S., Shi Y., Xie B., Xiang P., Cong B., Ma C., Wen D. (2024). Differential cannabinoid-like effects and pharmacokinetics of ADB-BICA, ADB-BINACA, ADB-4en-PINACA and MDMB-4en-PINACA in mice: A comparative study. Addict. Biol..

[24] Zhang H., Gao N., Tian X., Liu T., Fang Y., Zhou J., Wen Q., Xu B., Qi B., Gao J. (2015). Content and activity of human liver microsomal protein and prediction of individual hepatic clearance in vivo. Sci. Rep..

[25] Carlier J., Diao X., Scheidweiler K.B., Huestis M.A. (2017). Distinguishing Intake of New Synthetic Cannabinoids ADB-PINACA and 5F-ADB-PINACA with Human Hepatocyte Metabolites and High-Resolution Mass Spectrometry. Clin. Chem..

[26] Luo X., Huang Z., Huang K., Liu X., Yang N., Luo Q. (2024). Metabolic characteristic profiling of 1-amino-3,3-dimethyl-1-oxobutan-2-yl-derived indole and indazole synthetic cannabinoids in vitro. J. Pharm. Biomed. Anal..

[27] Bellec G., Goasduff T., Dreano Y., Menez J.F., Berthou F. (1996). Effect of the length of alkyl chain on the cytochrome P450 dependent metabolism of N-diakylnitrosamines. Cancer Lett..

